# Contribution of child health interventions to under-five mortality decline in Ghana: A modeling study using lives saved and missed opportunity tools

**DOI:** 10.1371/journal.pone.0267776

**Published:** 2022-08-01

**Authors:** Augusta Kolekang, Bismark Sarfo, Anthony Danso-Appiah, Duah Dwomoh, Patricia Akweongo

**Affiliations:** 1 School of Public Health, University for Development Studies, Tamale, Ghana; 2 School of Public Health, University of Ghana, Legon, Accra, Ghana; Njala University, SIERRA LEONE

## Abstract

**Background:**

Increased coverage of interventions have been advocated to reduce under-five mortality. However, Ghana failed to achieve the Millennium Development Goal on child survival in 2015 despite improved coverage levels of some child health interventions. Therefore, there is the need to determine which interventions contributed the most to mortality reduction and those that can further rapidly reduce mortality to inform the prioritization of the scale-up of interventions.

**Materials and methods:**

Deterministic mathematical modeling was done using Lives Saved and Missed Opportunity Tools. Secondary data was used, and the period of the evaluation was between 2008 and 2014. Some of the interventions assessed were complementary feeding, skilled delivery, and rotavirus vaccine.

**Results:**

A total of 48,084 lives were saved from changes in coverage of interventions and a reduction in the prevalence of stunting and wasting. Reduction in wasting prevalence saved 10,372(21.6%) lives, insecticide-treated net/indoor residual spraying 6,437(13.4%) lives saved, reduction in stunting 4,315(9%) lives saved and artemisinin-based combination therapy (ACTs) 4,325(9.0%) lives saved. If coverage levels of interventions in 2014 were scaled up to 90% in 2015, among neonates, full supportive care for prematurity (5,435 lives saved), full supportive care for neonatal sepsis/pneumonia (3,002 lives saved), and assisted vaginal delivery (2,163 lives saved), would have saved the most lives among neonates, while ACTs (4,925 lives saved), oral rehydration salts (ORS) (2,056 lives saved), and antibiotics for the treatment of pneumonia (1,805 lives saved) would have made the most impact on lives saved among children 1–59 months. Lastly, if all the interventions were at 100% coverage in 2014, the under-five mortality rate would have been 40.1 deaths per 1,000 live births in 2014.

**Discussion:**

The state of the package of interventions will likely not lead to rapid mortality reduction. Coverage and quality of childbirth-related interventions should be increased. Additionally, avenues to further reduce stunting and wasting, including increased breastfeeding and complementary feeding, will be beneficial.

## Introduction

Increased coverage of Maternal, Neonatal, Child Health, and Nutrition (MNCHN) interventions have been advocated during the period of the Millennium Development Goals (MDGs) and beyond that aimed to reduce under-five mortality (U5M) [1,2]. However, although Ghana achieved some increase in coverage of interventions during the period, it failed to achieve the MDGs target of a 75% decline in U5M between 1990 and 2015 [3] and is not on track to achieving the Sustainable Development Goal (SDG) 3.2 [4]. Ghana needed to achieve a U5M rate of about 40 deaths per 1,000 live births in 2015 to achieve the target, but U5M in 2014 was about 60 deaths per 1,000 live births [3]. Also, the Greater Accra and Ashanti Regions of Ghana, which had the lowest mortality rates historically, had the slowest U5M rate decline of 62 to 47 deaths per 1,000 live births and 78.2 to 80 deaths per 1,000 live births, respectively, compared to the national rate decline of 108 to 60 deaths per 1,000 live births between 1998 and 2014 [3,5]. These point to a possible stagnation and increase in mortality in the future. Progress in reducing neonatal deaths also lags compared to deaths after the neonatal period, with reports of an increasing proportion of neonatal deaths in some countries [6,7]. This calls for the prioritization of interventions implemented to scale-up high-impact interventions for maximum impact on U5M decline.

One of the earliest efforts at implementing interventions for the prevention of child mortality started in Ghana in 1978 with the introduction of the Expanded Programme on Immunization (EPI) [8]. Since then, several interventions have been introduced, and strategies to increase coverage of interventions implemented. The most recent addition to the pool of interventions was the malaria vaccine introduced in 2019 [9]. Vaccines had the highest coverage levels among the interventions being implemented that can affect U5M in Ghana. In contrast, water and sanitation interventions had the lowest coverage levels according to the 2014 Ghana Demographic and Health Survey (GDHS).

The Community-based Health Planning Services (CHPS) and the National Health Insurance Scheme (NHIS) are some strategies to increase access to healthcare services and, consequently, the coverage levels of interventions in Ghana [10]. However, increased coverage of interventions will not yield the needed mortality decline if implemented interventions are ineffective [11]. Differences in the impact of these interventions are expected with differences in the prevalence of diseases, risk factors, and over time. With the slow pace of progress in child survival in Ghana, there is the need to assess how these interventions impact child survival. The objective of this study was to evaluate the contribution of the various child health interventions to mortality decline between 2008 and 2014 and those with the potential to further contribute to mortality reduction if their coverage levels are increased.

## Materials and methods

### Study design

Deterministic mathematical modeling was done using Lives Saved Tool (LiST) and Missed Opportunity Tool (MOT) [12]. A demographic module (DemPro), family planning module (FamPlan), and AIDS impact module (AIM) were used as part of the LiST analysis. These tools are part of a suite of policy modeling tools in the spectrum software available at www.avenirhealth.org. Version 6.08 was used.

The LiST is a multi-cause deterministic mathematical population-level modeling tool used for evaluation and attribution. It can be used to evaluate the impact of multiple interventions or programmes implemented simultaneously. There were over 70 interventions in LiST at the global level as of 2011. The LiST has been validated and used in studies in several countries including Ghana [13–19]. It was used to model the number of lives saved and percent mortality rate reduction based on changes in intervention coverage levels and the decrease in the prevalence of stunting and wasting in this study [20–23].

In general, if coverage levels increase for the interventions, children who were previously unprotected from death due to lack of use of these interventions become protected. Therefore, additional lives are saved, or excess deaths are prevented due to the interventions relative to the previous coverage levels. Conversely, if coverage levels are reduced, it results in excess deaths, while no change in coverage results in zero lives saved. The interventions used in this analysis were those with coverage level information in the LiST. For stunting and wasting, a decrease in their prevalence reduces the risk of infections such as malaria, diarrhoea, and pneumonia and, consequently, deaths from these conditions.

The MOT determines the interventions that will make the most impact if intervention coverage levels were individually scaled up to 90%, one at a time, one year from the baseline year [12]. It, therefore, estimates the future potential impact of interventions, and thus, those to focus on to accelerate mortality decline. Although 90% is the default coverage level in the missed opportunity tool, it is also the target coverage level of vaccines and other interventions for the Ghana Health Service [24].

### Sources of data

The lives saved tool contains default data of several countries from different sources, including household surveys such as the Demographic and Health Surveys (DHS), Multiple Indicator Cluster Survey (MICS), and Malaria Indicator Survey (MIS), the World Health Organization (WHO), and the United Nations Children Emergency Fund (UNICEF). Coverage of maternal and child health intervention data comes from surveys such as DHS, MICS, and MIS, apart from vaccines from the WHO/UNICEF. Water and sanitation-related interventions come from the WHO/UNICEF Joint Monitoring Program for water supply and sanitation (WHO/UNICEF-JMP). Mortality rate data comes from the UN Inter-agency Group for Child Mortality Estimation (IGME). Coverage levels of some interventions for which data is not available are modeled using other intervention coverage levels, or assumptions are made on their coverage levels. Appendix A contains interventions in LiST on which data is available in Ghana.

### Lives saved tool modeling strategy

The lives saved tool makes a projection of the population under study using the DemPro (demographic projection) and calculates births and deaths that should occur in that population. The effect of family planning services on the number of births is applied using information from the FamPlan module. At the same time, the effect of AIDS on mortality is also applied using information from the AIM (AIDS impact module). With the number of deaths estimated, coverage of interventions to reduce mortality is applied to the population. Based on the effectiveness of the intervention(s) delivered in that population, the projected deaths are expected to change relative to the changes in coverage of the interventions since the interventions are expected to reduce the number of deaths that should occur in the population.

The basic formula for calculating the impact is impact = change in intervention coverage X effectiveness of intervention X affected fraction. Adjustments are made to this basic formula to account for previous lives saved and the herd effect of vaccines and bed net. Details of the modeling strategy are built in the LiST and published [25].

Intervention effectiveness is effectiveness established at the global or regional levels. It is in the LiST, while the affected fraction is the fraction of cause-specific mortality amenable to the particular intervention which is also in the tool [25].

The timeframe for the modeling was from the year 2008 to 2014. 2008 and 2014 were chosen because they are closest to the end of the Millennium Development Goals (MDGs) and the beginning of the Sustainable Development Goals (SDGs). Therefore, information from the study will provide an understanding of which interventions to focus on during the SDGs period to achieve rapid mortality reduction among children under five years old. The definitions of the interventions evaluated in this study are found in the LiST.

### Missed opportunity tool modeling strategy

The MOT estimates lives saved by automatically scaling up interventions with coverage levels below 90% to 90%, one intervention at a time. Interventions with coverage levels at or greater than 90% are excluded. Interventions with the most lives saved are those likely to contribute to lives saved/mortality reduction if their coverage levels are increased.

### Study area

The study was conducted in Ghana, a country with stable democratic governance since 1992. Healthcare expenditure in 2014 was 60 United States Dollars per capita [26]. Schieber, Cashin [10] has documented that Ghana’s U5M rate is high compared to its high healthcare spending. Related to this is Ghana’s slow progress made on under-five mortality during the period of the Millennium Development Goals (MDGs) [6].

### Study variables

Information in the LiST used were mortality rates (neonatal, infant, and under-five), coverage of interventions, stunting, wasting, and contraceptive prevalence. The outputs were mortality rates (neonatal and under-five) and lives saved. Neonatal mortality rate is the probability of dying within the first month of life. In contrast, under-five mortality is the probability of dying between birth and exactly five years [3]. The interventions included were iron intake, intermittent preventive treatment of malaria in pregnancy, tetanus toxoid vaccine (neonatal tetanus protection), skilled delivery, clean postnatal care, early initiation of breastfeeding, water connection in the home, time to improved water source, improved sanitation and insecticide-treated net/indoor residual spraying. From the LiST, at baseline (2008), the neonatal mortality rate was 31.2 deaths per 1,000 live births, while the under-5 mortality rate was 75.3 deaths per 1,000 live births. At the end line (2014), the neonatal mortality rate was 26.6 deaths per 1,000 live births, while the under-5 mortality rate was 57.1 deaths per 1,000 live births in Ghana.

### Definition of some of the interventions

**Caesarean delivery:** percent of women requiring surgical intervention if indicated (emergency caesarean birth for obstructed labour, uterine rupture, or foetal distress).

**Complementary feeding:** percent of 6–23-month-old children receiving minimum dietary diversity (4+ food groups).

**Case management of severe neonatal infection (sepsis/pneumonia):** sum of the three levels of case management for severe infection in the neonatal period: oral antibiotics, injectable antibiotics, and full supportive care.

**Full supportive care for neonatal sepsis/pneumonia:** full supportive care for neonatal sepsis/pneumonia.

**Artemisinin compounds for the treatment of malaria:** percent of children treated within 48 hours of the onset of fever in malaria-endemic areas with an artmesinin-containing compound (artemisinin-based combination therapy).

### Data analysis

The default data of Ghana in the LiST was loaded into the tool, and the analyses done. First of all, to assess the contribution of only interventions to mortality decline between 2008 and 2014, the baseline for the impact evaluation was set at 2008 and the end line in 2014. Then, interpolation was done between 2008 and 2014 (linear scale-up of coverage of interventions between the baseline and end-line), and lives saved by interventions between 2008 and 2014 were reported. Finally, changes were made to this configuration (modeling strategy) to allow for estimates of lives saved, and mortality rate reduced based on the direct entry of stunting and wasting (reduction in the prevalence of stunting and wasting between 2008 and 2014) in addition to the interventions. Lives saved due to changes in intervention coverage and differences in the prevalence of stunting and wasting were reported.

Secondly, to assess the interventions with the most potential to reduce mortality if coverage levels were scaled up individually to 90% from the 2014 coverage levels, a new projection was created with 2014 as the baseline and 2019 (a minimum of 5 years is allowed to estimate impact using the LiST) the end line. The 2014 baseline projection was then uploaded into the missed opportunity tool, and lives saved by each intervention were reported. All analyses were done in duplicates, and the results were the same.

Lastly, for the analysis to assess the impact of interventions at scale-up of coverage levels to 100% in 2014, the projection created with 2008 as the baseline and 2014 as the end line was used. Next, all the coverage levels of interventions with available data in 2014 were changed to 100%, interpolation done and lives saved, mortality reduction and neonatal and under-five mortality rates reported. One hundred percent (100%) was chosen because coverage levels of DPT, pneumococcal, rotavirus, and measles vaccines in the LiST were over 90%.

#### Ethics issues and consent to participate

Publicly available data was used, and the study did not involve human subjects since secondary data was used.

## Results and discussion

### Results

A total of 48,084 lives were saved (Table 1), resulting from changes in coverage levels of interventions, and reduced stunting and wasting among children born between 2003 and 2014. In addition, modeling involving only the interventions resulted in 34,477 lives saved/additional deaths prevented.

**Table 1:** Additional child lives saved and mortality reduction by intervention between 2008 and 2014 among children 0–59 months.

Interventions				Lives saved	
Pregnancy	Baseline (2008) coverage	End line (2014) coverage	Coverage change	Intervention based modeling	Direct entry of stunting and wasting
Tetanus toxoid vaccination (TT)	86.0	88.0	2.0	95(0.3)	95(0.2)
Prevention of malaria in pregnancy	43.7	67.5	23.8	810(2.3)	777(1.6)
Syphilis detection and treatment	23.6	24.0	0.4	4(0.0)	4(0.0)
Prevention of mother-to-child transmission of HIV (including breastfeeding choices) (PMTCT)	0.0	23.3	23.3	1,445(4.2)	1,446(3.0)
Maternal age and birth order				2(0.0)	2(0.0)
**Childbirth**					
Clean birth environment	46.8	59.9	13.1	736(2.1)	736(1.5)
Immediate drying and additional stimulation	52.3	66.9	14.6	809(2.3)	808(1.7)
Thermal protection	56.5	72.2	15.8	1,205(3.5)	1,205(2.5)
Clean cord care	54.5	69.7	15.2	1,318(3.8)	1,318(2.7)
Antibiotics for preterm or prolonged premature rupture of the membranes (PROM)	42.7	54.7	11.9	320(0.9)	320(0.7)
Parenteral administration of antibiotics	42.7	54.7	11.9	320(0.9)	320(0.7)
Assisted vaginal delivery	14.4	18.5	4.0	439(1.3)	439(0.9)
Neonatal resuscitation	31.4	40.2	8.8	988(2.9)	988(2.1)
Caesarean delivery	48.3	76.4	28.1	4,145(12.0)	4,145(8.6)
**Breastfeeding**					
Age-appropriate breastfeeding practices	60.6	50.0	-10.6	-1,711(-5.0)	-1,786(-3.7)
**Preventive**					
Change in stunting prevalence	27.5	18.8	8.7	NA	4,315(9.0)
Vitamin A supplementation (2 doses)	24.0	23.0	-1.0	-21(-0.1)	-21(0.0)
Basic sanitation	12.7	16.5	3.8	120(0.3)	115(0.2)
Point-of-use filtered water	0.9	0.8	-0.1	-7(0.0)	-7(0.0)
Piped water	39.4	35.3	-4.2	-191(-0.6)	-190(-0.4)
Handwashing with soap	0.0	40.9	40.9	249(0.7)	234(0.5)
Insecticide-treated net/indoor residual spraying (ITN/IRS)	41.7	70.9	29.2	6433(18.7)	6437(13.4)
Complementary feeding (via reduction in stunting)	42.5	24.4	-18.1	394(1.1)	NA
Complementary feeding (via reduction in wasting)	42.5	24.4	-18.0	428(1.2)	NA
**Vaccines**					
Diphtheria, pertussis, and tetanus (DPT) vaccine	88.0	98.0	10.8	869(2.5)	870(1.8)
*Haemophilus influenzae* type B	88.0	98.0	10.8	244(0.7)	243(0.5)
Pneumococcal vaccine (3 doses)	0.0	99.0	99.0	3,931(11.4)	3,938(8.2)
Rotavirus vaccine (3 doses)	0.0	98.0	98.0	655(1.9)	669(1.4)
Measles vaccine (1 dose)	89.8	92.0	5.4	239(0.7)	226(0.5)
**Curative after birth**					
Case management of neonatal sepsis/pneumonia	57.1	73.1	16.0	4,271(12.4)	4,271(8.9)
Oral rehydration salts (ORS)	44.5	48.6	4.1	985(2.9)	917(1.9)
Antibiotics for treatment of dysentery	46.4	42.2	-4.0	-116(-0.3)	-118(-0.2)
Zinc for treatment of diarrhoea	1.8	7.4	5.6	223(0.6)	208(0.4)
Oral antibiotics for pneumonia	50.7	52.6	1.9	422(1.2)	396(0.8)
Vitamin A for treatment of measles	24.0	23.0	-1.0	-9(0.0)	-9(0.0)
Artemisinin-based combination therapy (ACTs) (within 48hours)	11.3	26.2	14.9	4,322(12.5)	4,325(9.0)
Treatment for severe acute malnutrition (SAM)	0.0	2.7	2.7	37(0.1)	0(0.0)
Change in wasting prevalence	8.6	4.7	3.9	NA	10,372(21.6)
Cotrimoxazole	2.7	4.1	1.4	31(0.1)	31(0.1)
ART for children	7.4	22.7	15.2	43(0.1)	45(0.1)
Total				34,477(100)	48,084(100)

Interventions that saved the most lives considering the effect of reduction in the prevalence of stunting and wasting among children under-five years were ITN/IRS, 6,437 (13.4%) lives saved, ACTs, 4,325 (9.0%) lives saved, case management of neonatal sepsis/pneumonia, 4,271(8.9%) lives saved, caesarean delivery, 4,145(8.6%) lives saved, and pneumococcal vaccine, 3,938 (8.2%) lives saved (Table 1). Reduction in wasting saved 10,372 (21.6%), while a decrease in the prevalence of stunting also saved 4,315 (9.0%) lives. On the contrary, breastfeeding, -1,786(-3.7.0%), piped water, -190 (-0.4%), antibiotics for treatment of dysentery, -118 (-0.2%), and vitamin A supplementation, -21 (-0.0%) resulted in negative lives saved (excess deaths/additional deaths) between 2008 and 2014 (Table 1).

If coverage levels of interventions in 2014 were scaled-up to 90% in 2015, each intervention at a time, full supportive care for prematurity (5,435 lives saved), full supportive care for neonatal sepsis/pneumonia (3,002 lives saved), assisted vaginal delivery (2,163 lives saved) and kangaroo mother care (2,010 lives saved) would have saved the most lives among neonates (Table 2). In addition, ACTs (4,925 lives saved), ORS (2,056 lives saved), antibiotics for the treatment of pneumonia (1,805 lives saved), and prevention of mother-to-child transmission of HIV (including breastfeeding choices) (1,020 lives saved) would have made the most impact on mortality reduction among children 1–59 months (Table 2).

**Table 2:** Lives saved at 90% intervention coverage level, one intervention at a time among children 0–59 months.

Intervention	Baseline coverage	Total additional deaths prevented	
		0–1 months	1–59 months
Full supportive care for prematurity	0.0	5,435	0
Artemisinin compounds for treatment of malaria (ACT)	26.2	0	4,925
Full supportive care for neonatal sepsis/pneumonia	0.0	3,002	0
Assisted vaginal delivery	18.5	2,163	0
Oral rehydration solution (ORS)	48.6	64	2,056
Kangaroo mother care (KMC)	0.0	2,010	0
Oral antibiotics for pneumonia	52.6	0	1,805
Neonatal resuscitation	40.2	1,582	0
Injectable antibiotics for neonatal sepsis	73.1	1,382	0
Prevention of mother-to-child transmission of HIV (including breastfeeding choices) (PMTCT)	23.3	0	1,020
Households protected from malaria (ITN/IRS)	70.9	0	999
Multiple micronutrient supplementation in pregnancy	0.0	871	18
Treatment for moderate acute malnutrition (MAM)	0.0	0	849
Point-of-use filtered water	0.8	22	781
Zinc supplementation	0.0	0	785
Zinc for the treatment of diarrhoea	7.4	20	642
Oral antibiotics for neonatal sepsis	0.0	655	0
Cesarean delivery	76.4	653	0
Breastfeeding promotion	50.0	93	511
Clean cord care	69.7	515	0
Piped water	35.3	14	497
Cotrimoxazole	4.1	0	485
Clean birth environment	59.9	454	0
Basic sanitation	16.5	12	437
Thermal protection	72.2	398	0
Immediate drying and additional stimulation	66.9	360	0
Vitamin A supplementation	23.0	0	304
Treatment for severe acute malnutrition (SAM)	2.7	0	289
Antibiotics for preterm or prolonged premature rupture of the membranes (PROM)	54.7	265	0
Parenteral administration of antibiotics	54.7	265	0
Antibiotics for treatment of dysentery	42.4	7	239
Prevention of malaria in pregnancy	67.5	223	15
Antiretroviral therapy (ART)	22.7	0	236
Appropriate complementary feeding	24.4	0	219
Syphilis detection and treatment	24.0	219	0
Folic acid fortification	0.0	207	0
Calcium supplementation	0.0	186	1
Handwashing with soap	40.9	4	128
Balanced energy supplementation	0.0	61	2
Vitamin A for treatment of measles	23.0	0	32
Tetanus toxoid vaccination (TT)	88.0	21	0
Measles vaccine	92.0	0	2

Also, if coverage levels of all the interventions were scaled up to 100% in 2014, the neonatal mortality rate would have been 21.7 deaths per 1,000 live births instead of 26.2 deaths per 1,000 live births, while the U5M rate would have been 40.1 deaths per 1,000 live births instead of 57.1 deaths per 1,000 live births in 2014. Under this scenario (coverage of interventions at 100%), results from the effect of only interventions showed that ACTs, 21,204 (19.6%), ITN/IRS, 12,906 (11.9%), oral antibiotics for pneumonia, 9,173 (8.5%) and age-appropriate breastfeeding practices, 7,935 (7.3%) would have contributed the most to mortality reduction (Table 3).

**Table 3:** Additional child lives saved by intervention in 2014 intervention coverage levels were scale-up to 100% among children 0–59 months.

Intervention	Coverage increased from baseline to 100%	Lives saved (%)
Tetanus toxoid vaccination (TT)	14.0	669(0.6)
Prevention of malaria in pregnancy	56.3	1,896(1.7)
Syphilis detection and treatment	1.1	15(0.0)
Prevention of mother-to-child transmission of HIV (including breastfeeding choices) (PMTCT)	100.0	1,450(1.3)
Maternal age and birth order		2(0.0)
**Childbirth**		
Clean birth environment	35.2	1,850(1.7)
Immediate drying and additional stimulation	39.3	2,078(1.9)
Thermal protection	42.4	3,026(2.8)
Clean cord care	40.9	3,041(2.8)
Antibiotics for preterm or prolonged premature rupture of the membranes (PROM)	32.1	836(0.8)
Parenteral administration of antibiotics	32.1	836(0.8)
Assisted vaginal delivery	10.8	1,104(1.0)
Neonatal resuscitation	23.6	2,511(2.3)
Parenteral administration of uterotonics	38.3	0(0.0)
Cesarean delivery	51.7	7,134(6.6)
**Breastfeeding**		
Age-appropriate breastfeeding practices	50.0	7,935(7.3)
**Preventive**		
Vitamin A supplementation	76.0	1,247(1.2)
Basic sanitation	87.3	1,992(1.8)
Point-of-use filtered water	99.1	5,560(5.1)
Piped water	60.6	2,140(2.0)
Handwashing with soap	100.0	387(0.4)
Hygienic disposal of children’s stools	57.6	0(0.0)
Households protected from malaria (ITN/IRS)	58.3	12,906(11.9)
Complementary feeding (via reduction in stunting)	57.5	1,361(1.3)
Complementary feeding (via reduction in wasting)	57.5	464(0.4)
**Vaccines**		
DPT vaccine	12.0	882(0.8)
*Haemophilus influenzae* b vaccine	12.0	222(0.2)
Pneumococcal vaccine (3 doses)	100.0	3,433(3.2)
Rotavirus vaccine (3 doses)	100.0	416(0.4)
Measles vaccine (1 dose)	10.2	970(0.9)
**Curative after birth**		
Case management of neonatal sepsis/pneumonia	16.0	3,617(3.3)
Oral rehydration solution (ORS)	55.5	4,911(4.5)
Antibiotics for treatment of dysentery	53.6	540(0.5)
Zinc for the treatment of diarrhea	98.2	1,438(1.3)
Oral antibiotics for pneumonia	49.3	9,173(8.5)
Vitamin A for treatment of measles	76.0	237(0.2)
Artemisinin compounds for the treatment of malaria (ACT)	88.7	21,204(19.6)
Treatment for severe acute malnutrition (SAM)	100.0	830(0.8)
Cotrimoxazole	97.3	31(0.0)
Antiretroviral therapy (ART)	92.6	46(0.0)
Total		108,390(100)

### Discussion

Ghana failed to achieve the MDG4 target despite efforts at increasing intervention coverage levels. This study, therefore, evaluated the contribution of the various interventions to mortality decline. Information from the study will help prioritize intervention scale-up. Reduction in the prevalence of wasting and stunting, malaria control interventions (ACT and ITN/IRS), pneumococcal vaccines, and childbirth-related interventions (case management of neonatal sepsis/pneumonia, caesarean delivery, clean cord care, and thermal protection) caused mortality reduction. In contrast, nutrition (breastfeeding, vitamin A supplementation, and vitamin A for the treatment of measles) and water-related (piped water and point-of-use filtered water) interventions were responsible for excess mortality between 2008 and 2014.

Proper maternal and child nutrition plays a crucial role in child survival. It reduces the risk of important causes of death such as malaria, diarrhoea, and pneumonia [6,7] and risk factors such as preterm, small for gestational age, stunting, and wasting [3,27]. Unfortunately, breastfeeding and complementary feeding prevalence reduced between 2008 and 2014 among children under-five years in Ghana [3,28]. This resulted in -1,786 (-3.7%) excess deaths due to a reduction in breastfeeding (60.6% to 50%) between 2008 and 2014. Despite this, a decline in stunting (28.0% to 18.8%) and wasting (8.5% to 4.7%) was achieved between 2008 and 2014, contributing to a 9.0% and 21.6% mortality reduction, respectively. The food fortification programme with vitamin A, the Livelihood Empowerment Against Poverty (LEAP) programme introduced in 2007, the school feeding programme introduced in the 2005–2006 academic year [29], and poverty reduction during the period of evaluation could explain the decrease in stunting and wasting despite a reduction in the prevalence of complementary feeding [3,28]. It implies that further mortality decline can be achieved if complementary feeding and breastfeeding coverage levels are increased. Similar results of reducing stunting and wasting to mortality decline have been reported [13,30–32].

Generally, negative lives are saved if the coverage level of an intervention is reduced during the evaluation period. This means taking the population that needed to be protected by the intervention, the percentage protected was less at the end line than the baseline. Therefore, a proportion of the people became unprotected and died as a result. It could be the case that the intervention is available but not all the population that needs it uses it. Examples of such interventions are breastfeeding, vitamin A supplementation and antibiotics to treat dysentery. The negative lives saved could also be due to an increase in the population size not matched with the scale-up of the intervention. This could be the case with piped water. Population growth is not commensurate with the provision of piped water.

Malaria remains a top cause of under-five deaths in Ghana, and therefore, effective interventions targeting malaria deaths should have a high impact. While household ITN/IRS coverage made a substantial impact (15% mortality reduction), they still have the potential for increased impact if coverage is scaled up. Similar results of the effect of bed net on mortality have been documented [13,17,30,33]. Comparing ACTs to ITN/IRS, ACTs also saved many lives (9% reduction in mortality rate), but this was lower than that achieved by ITN/IRS (13.4% reduction in mortality rate). This is likely due to the low coverage change of ACTs (11.3% to 26.6%) between 2008 and 2014 compared to ITN/IRS (41.7% to 70.9%). This low coverage could explain the continuously enormous contribution of malaria to U5M and the generally low mortality decline in Ghana since uncomplicated malaria can progress rapidly to severe disease and death due to delay in treatment. It also implies that ACT can save additional lives since its current coverage level is low in the country. Therefore, it should be one of the interventions to focus on to further reduce mortality, especially in malaria high burdened regions of Ghana.

Vaccines have historically, and in recent times, contributed greatly to mortality reduction [17,30,31,34]. In this study, pneumococcal and rotavirus vaccines greatly impacted mortality reduction, 8% and 3%, respectively. These vaccines are relatively new, were introduced in 2012, accounting for their high coverage level change, 0 to 99% for pneumococcal vaccine and 0 to 98% for rotavirus vaccine between 2008 and 2014 [3,8]. However, they are not likely to save more lives than their current impact in the future, just like the old vaccines. This is because, with a national level coverage of over 90% for most vaccines, any further increase in coverage will be less than 10%. Also, if the high coverage levels of these vaccines reduce the prevalence of vaccine-preventable diseases in the population like diphtheria and tetanus, they will likely not contribute greatly to mortality decline even if their coverage levels are further increased since very few children will suffer from these conditions. Considering that one of the most effective tools for reducing mortality does not have much potential for further mortality reduction, coverages of other high-impact interventions, especially those with low coverage levels need to be increased. The recently introduced malaria vaccine might contribute to filling this gap [9].

In this study, interventions related to childbirth, such as caesarean delivery delivered by skilled personnel, contributed the most to lives saved (8.6% of lives saved). Skilled delivery is one of the main strategies to reduce neonatal mortality, an important contributor to U5M. In Tanzania, skilled delivery and emergency obstetric care among neonates contributed to a 29% (6,200 lives saved) reduction in mortality between 2000 and 2012 [30]. However, Ethiopia achieved the MDG4 target on child survival with skilled delivery coverage between 2000 and 2011 of less than 5%, contributing to a 3% decline in U5M [31]. The quality of skilled delivery has been questioned, and neonatal mortality ranked second in deaths due to poor quality of care [35].

In Ghana, while the coverage level of skilled delivery was 84.3% in 2008 and 92.1% in 2014 in the Greater Accra Region, neonatal mortality increased from 21 deaths per 1,000 live births to 25 deaths per 1,000 live births between 2008 and 2014. Also, in the Ashanti Region, skilled delivery coverage was 72.6% in 2008 and 86.3% in 2014; still, the region recorded an increase in neonatal mortality from 35 deaths per 1,000 live births to 42 deaths per 1,000 live birth from 2008 to 2014 [3,28]. On the contrary, in the Upper West Region, where the skilled delivery coverage level was 46.1% in 2008 and 63.7% in 2014, neonatal mortality decreased from 45 deaths per 1,000 live births to 24 deaths per 1,000 live births between the periods. Likewise, in the Northern Region where skilled delivery coverage was 27.2% in 2008 and 36.4% in 2014, neonatal mortality declined from 35 deaths per 1,000 live births to 24 deaths per 1,000 live births within the period [3,28].

#### The trajectory of under-five mortality in Ghana

In 2014, if coverage levels of all the interventions were scaled up to 100%, the neonatal mortality rate would have declined from 31.2 deaths per 1,000 live births to 21.7 deaths per 1,000 live births, while the U5M rate would have declined from 75.3 deaths per 1,000 live births to 40.1 deaths per 1,000 live births. Although this would have been daunting and impossible to achieve, Ghana would have achieved the MDGs target of an under-five mortality rate of at most 40 deaths per 1000 live births. However, any further rapid decrease in mortality will be impossible once the coverage levels of most interventions reach 100%. This suggests that the available packages of care remain suboptimal even if they were taken to 100% coverage; hence, the need to continue innovating in alternative complementary interventions. This situation is consistent with the documented slow decline in child mortality in Ghana [6]. This slow decline could mean that the interventions implemented are not producing the needed reduction in mortality, and the quality of the interventions could be the cause [35,36].

If this trend persists, Ghana will not achieve the SDGs targets of neonatal mortality of 12 deaths per 1,000 live births and U5M of 25 deaths per 1,000 live births as already projected [4]. The mortality rate will likely be stagnant, as observed among regions in Ghana with historically low under-five mortality rates [3]. Historically, vaccines have contributed greatly to mortality reduction, but future mortality decline is unlikely due to their current high (over 90%) coverage levels [3]. Therefore, most vaccines are not part of the interventions evaluated that can further reduce mortality in Ghana. Also, neonatal mortality contributes greatly to U5M. From assessing the interventions that can further reduce neonatal mortality if coverage levels were increased, interventions that operate at childbirth related to skilled delivery are the most important. Unfortunately, access to quality skilled care remains a challenge. It, therefore, suggests that rapid mortality decline will only be possible if there is an increase in coverage and quality of the few high-impact interventions related to nutrition, water, hygiene and childbirth.

On study limitations and strengths, LiST assumes constant effectiveness of interventions that might not always hold because several factors, including the quality of the interventions and their implementation, could affect interventions when implemented under real-life conditions [37]. Furthermore, data is not available for some interventions in LiST, and assumptions are made on these. However, validation studies show that LiST produces reliable estimates based on the information available. Additionally, the LiST assumes that distal factors like household factors such as wealth, maternal education, and place of residence affect mortality through interventions that might be problematic [23]. Validation studies have been performed, and the tool has been found to work well [15,16,18,38–41]. Lastly, this analysis used only data contained in the LiST, and it did not consider the feasibility of the scale-up scenarios created.

In conclusion, this study assessed the impact of interventions on under-five mortality. Insecticide-treated net/indoor residual spraying, ACT, pneumococcal vaccine, rotavirus vaccine, skilled delivery, and reduction in stunting and wasting made the most impact on mortality among children born between 2003 and 2014 in Ghana. Childbirth-related interventions, ITN/IRS, ACT, and water and sanitation interventions would have made the most impact if the 2014 coverages of interventions were scaled up to 90% in 2015. Despite the potential impact of the current package of interventions, if coverage levels are increased, this increase might not rapidly reduce mortality. Avenues to reduce stunting and wasting, including increasing breastfeeding and complementary feeding, will be beneficial. Mothers and caregivers should be encouraged to provide balanced diets for their children. Together with the Ministry of Health and Ghana Health Service, the Ministry of Education should ensure that meals provided under the school feeding programme are balanced diets.

## Supporting information

S1 Table All interventions used in the modeling. (DOCX)

S2 Table Interventions that were scaled up to 100%. (DOCX)
