# Supplementary material for: Contribution of child health interventions to under-five mortality decline in Ghana: A modeling study using lives saved and missed opportunity tools
Source: PLoS One. 2022 Aug 1;17(8):e0267776. doi: 10.1371/journal.pone.0267776 (PMC9342718; doi:10.1371/journal.pone.0267776)
Supplement: S1 Table — (DOCX) [file pone.0267776.s001.docx]

Table 1: All interventions used in the modeling

| Full supportive care for prematurity |
| --- |
| Artemisinin compounds for the treatment of malaria (ACT) |
| Full supportive care for neonatal sepsis/pneumonia |
| Assisted vaginal delivery |
| Oral rehydration solution (ORS) |
| Kangaroo mother care (KMC) |
| Oral antibiotics for pneumonia |
| Neonatal resuscitation |
| Injectable antibiotics for neonatal sepsis |
| Prevention of mother-to-child transmission of HIV (including breastfeeding choices) (PMTCT) |
| Households protected from malaria (ITN/IRS) |
| Multiple micronutrient supplementation in pregnancy |
| Treatment for moderate acute malnutrition (MAM) |
| Point-of-use filtered water |
| Zinc supplementation |
| Zinc for the treatment of diarrhoea |
| Oral antibiotics for neonatal sepsis |
| Cesarean delivery |
| Breastfeeding promotion |
| Clean cord care |
| Piped water |
| Cotrimoxazole |
| Clean birth environment |
| Basic sanitation |
| Thermal protection |
| Immediate drying and additional stimulation |
| Vitamin A supplementation |
| Treatment for severe acute malnutrition (SAM) |
| Antibiotics for preterm or prolonged premature rupture of the membranes (PROM) |
| Parenteral administration of antibiotics |
| Antibiotics for treatment of dysentery |
| Prevention of malaria in pregnancy |
| Antiretroviral therapy (ART) |
| Appropriate complementary feeding |
| Syphilis detection and treatment |
| Folic acid fortification |
| Calcium supplementation |
| Handwashing with soap |
| Balanced energy supplementation |
| Vitamin A for treatment of measles |
| Tetanus toxoid vaccination (TT) |
| Measles vaccine |
| Diphtheria, Pertussis, and Tetanus (DPT) vaccine |
| *Haemophilus influenzae type* B |
