# Supplementary material for: Contribution of child health interventions to under-five mortality decline in Ghana: A modeling study using lives saved and missed opportunity tools
Source: PLoS One. 2022 Aug 1;17(8):e0267776. doi: 10.1371/journal.pone.0267776 (PMC9342718; doi:10.1371/journal.pone.0267776)
Supplement: S2 Table — (DOCX) [file pone.0267776.s002.docx]

Table 2: Interventions that were scaled up to 100%

| Antenatal care visits (at least one visit) |
| --- |
| Antenatal care visits (at least 4 visits) |
| Tetanus toxoid vaccination (TT) |
| Prevention of malaria in pregnancy |
| Iron intake |
| Health facility delivery |
| Cesarean delivery |
| Breastfeeding |
| Exclusive breastfeeding among children less than 1 month |
| Exclusive breastfeeding among children 1 to 5 months |
| Any breastfeeding among children 6 months to 11 months |
| Any breastfeeding among children 12 months to 23 months |
| Early initiation of breastfeeding (within 1 hour after delivery) |
| Complementary feeding (education only) |
| Complementary feeding (education and supplementation) |
| Vitamin A supplementation |
| Zinc supplementation |
| Basic sanitation |
| Point-of-use filtered water |
| Piped water |
| Handwashing with soap |
| Households protected from malaria (ITN/IRS) |
| Oral rehydration solution (ORS) |
| Antibiotics for the treatment of dysentery |
| Zinc for treatment of diarrhoea |
| Oral antibiotics for pneumonia |
| Vitamin A for treatment of measles |
| Artemisinin compounds for treatment of malaria (ACT) |
| Treatment for severe acute malnutrition (SAM) |
| Diphtheria, Pertussis, and Tetanus (DPT) vaccine |
| *Haemophilus influenzae* type B |
| Pneumococcal vaccine |
| Rota vaccine |
| Measles vaccine |

These interventions have coverage level information available in the Lives Saved Tool (LIST). Coverage level information was not assumed or modeled using other coverage level information.
